# Sebaceous lymphadenoma of the parotid: a rare case report of an entity mimicking other salivary tumors

**DOI:** 10.1093/jscr/rjaf382

**Published:** 2025-06-05

**Authors:** Amine Oussalem, Zein A B El Hassene, Bouchra Dani, Malik Boulaadas

**Affiliations:** Department of Maxillo-Facial Surgery, Avicenne Hospital, Area Lamfadel Cherkaoui, Rabat – Institut, BP 6527 Rabat, Morocco; Department of Maxillo-Facial Surgery, Avicenne Hospital, Area Lamfadel Cherkaoui, Rabat – Institut, BP 6527 Rabat, Morocco; Department of Maxillo-Facial Surgery, Avicenne Hospital, Area Lamfadel Cherkaoui, Rabat – Institut, BP 6527 Rabat, Morocco; Department of Maxillo-Facial Surgery, Avicenne Hospital, Area Lamfadel Cherkaoui, Rabat – Institut, BP 6527 Rabat, Morocco

**Keywords:** sebaceous lymphadenoma, parotid gland, salivary gland neoplasms, differential diagnosis, rare benign tumor

## Abstract

Sebaceous lymphadenoma is a rare benign tumor of the salivary glands, with fewer than 50 cases reported worldwide. Its clinical and radiological resemblance to malignant tumors poses diagnostic challenges. A 35-year-old female presented with a slow-growing, painless left parotid mass persisting for 2 years. Ultrasonography revealed a well-circumscribed, hypoechoic nodule measuring 2.5 cm. Fine-needle aspiration cytology suggested a benign lymphoid lesion, but definitive diagnosis required histopathological examination post-superficial parotidectomy. Microscopic analysis showed proliferating sebaceous cells within lymphoid stroma, confirmed by immunohistochemistry (EMA+, CK7–). No recurrence was observed at 12-month follow-up. This case underscores the importance of histopathology in distinguishing sebaceous lymphadenoma from carcinomas (e.g. sebaceous carcinoma) or Warthin tumor, particularly in regions with limited molecular diagnostic resources. Despite its rarity, sebaceous lymphadenoma should be considered in differential diagnoses of parotid masses to avoid unnecessary aggressive treatments.

## Introduction

Sebaceous lymphadenoma is a rare benign tumor of the salivary glands, first described by Smith *et al.* in 1950. It most frequently involves the parotid gland and is characterized histologically by well-circumscribed epithelial islands with sebaceous differentiation within a prominent lymphoid stroma [1, 2].

Due to its uncommon nature and overlapping clinical-radiological features with other benign or low-grade malignant salivary tumors, it often poses a diagnostic challenge [3, 4].

The differential diagnosis includes Warthin tumor, basal cell adenoma, pleomorphic adenoma, and low-grade mucoepidermoid carcinoma, which may share imaging similarities [5].

In most cases, diagnosis is only confirmed postoperatively through histopathological and immunohistochemical analysis [6].

We report a case of sebaceous lymphadenoma of the parotid gland in a 37-year-old male, presenting as a slowly enlarging, painless swelling of the left parotid region, and managed successfully by superficial parotidectomy with facial nerve preservation. This case contributes to the limited number of reports in the literature and aims to reinforce the importance of considering this rare entity in the differential diagnosis of parotid masses.

## Case report

**Figure 1:**
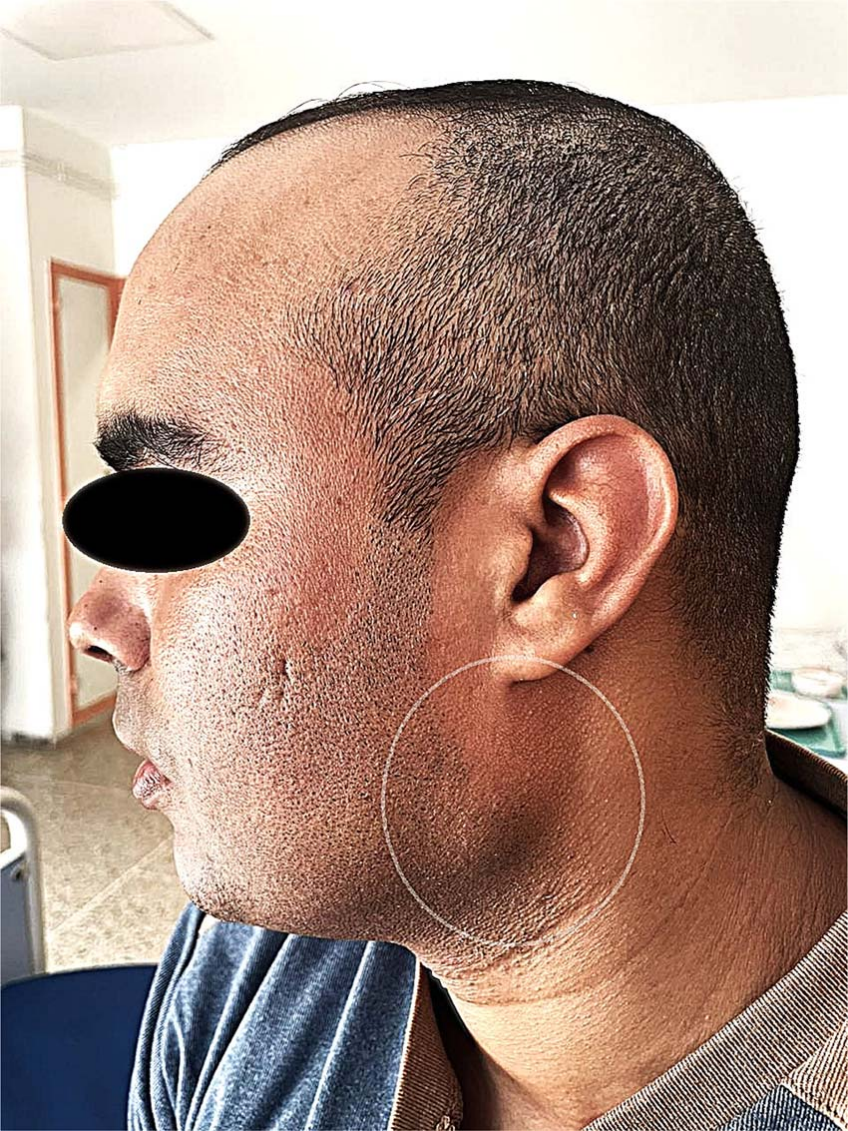
Preoperative photograph showing the sublobular swelling on the left side of the patient's face.

A 37-year-old male, with no significant past medical, family, psychosocial, genetic, and interventions history, presented with a progressively enlarging, painless swelling in the left sublobular parotid region that had been evolving over a 1-year period. There were no associated symptoms such as fever, weight loss, or facial weakness. Clinical examination revealed a firm, non-tender, mobile mass ⁓2 cm in diameter, located below the left ear lobe. The mass was not pulsatile and showed no overlying skin changes. There was no facial nerve dysfunction, no intraoral abnormalities, and no cervical lymphadenopathy. The Stensen’s duct appeared normal with no discharge of pus or blood (Fig. 1).

**Figure 2:**
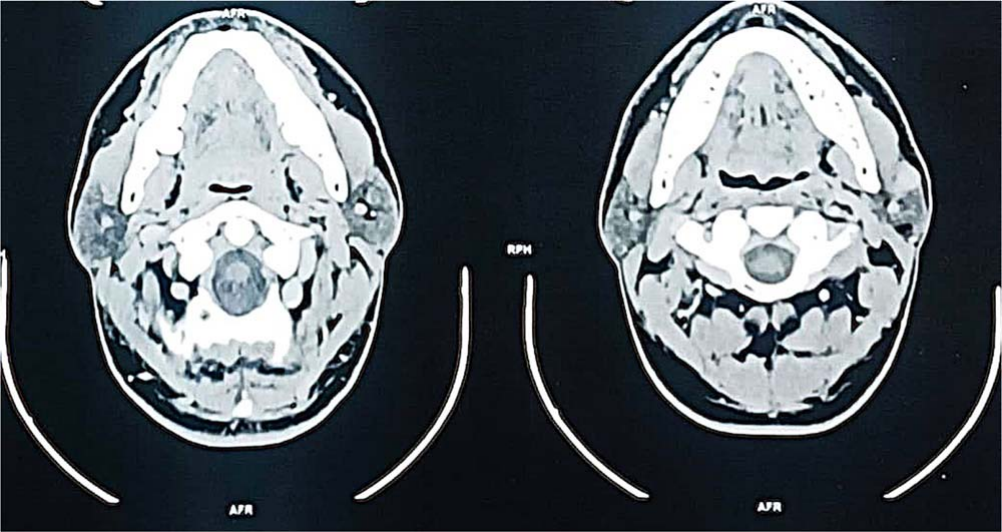
Axial contrast-enhanced CT scan demonstrating a well-defined, enhancing mass in the superficial lobe of the left parotid gland.

**Figure 3:**
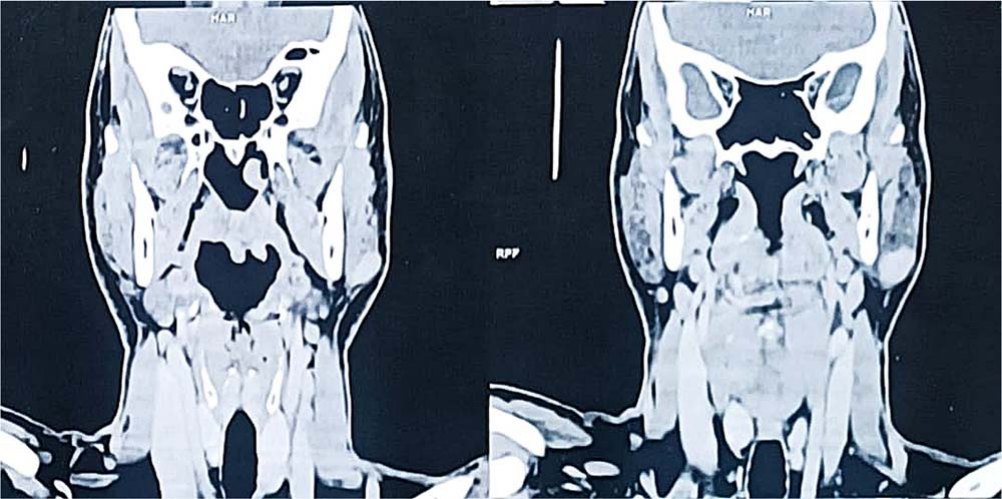
Coronal contrast-enhanced CT scan confirming the presence of an oval, homogeneous, enhancing lesion in the left parotid gland.

A contrast-enhanced computed tomography (CT) scan of the neck revealed a well-circumscribed, oval lesion located within the superficial lobe of the left parotid gland. The mass measured 20 × 16 × 14 mm, appeared isodense and homogeneous on pre-contrast images, and showed intense enhancement after contrast injection, consistent with a solid, vascular lesion (Figs. 2 and 3).

**Figure 4:**
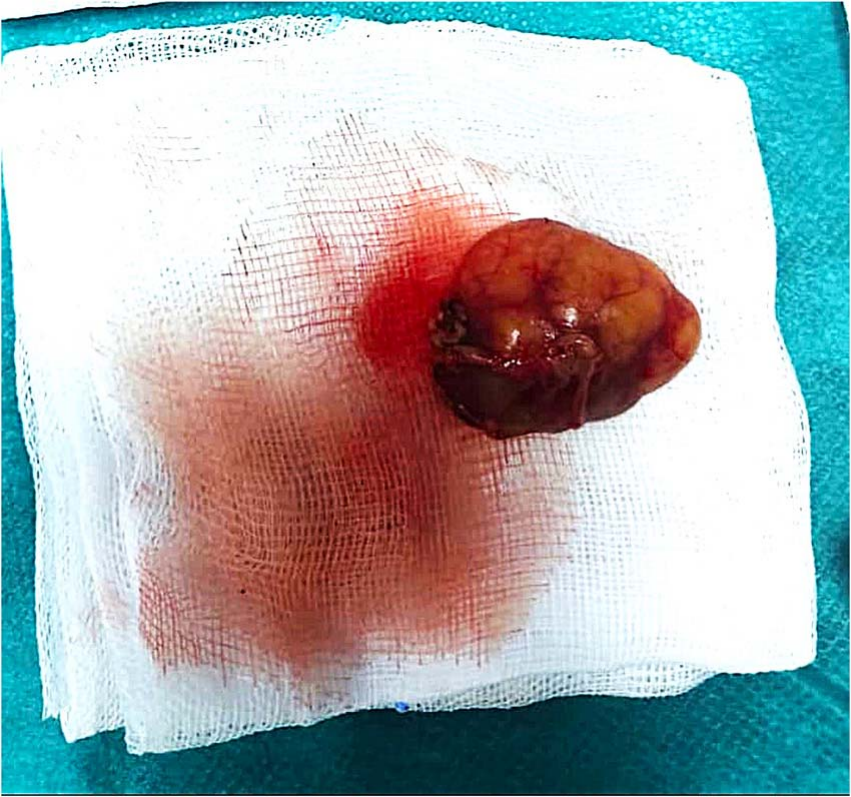
Macroscopic view of the excised tumor following superficial parotidectomy.

The patient underwent a left superficial parotidectomy under general anesthesia without the use of neuromuscular blockade. The challenge was to preserve all branches of the facial nerve. Intraoperatively, the tumor was well-encapsulated and completely excised with preservation of all branches of the facial nerve (Fig. 4).

Histopathological examination revealed a well-circumscribed tumor composed of sebaceous elements within a prominent lymphoid background, without cytological atypia or mitotic activity. Immunohistochemistry supported the diagnosis of sebaceous lymphadenoma and elimited pleomorphe adenoma, who was the differential diagnosis.

The postoperative course was uneventful, with no unanticiped events and a good adherence and tolerability from the patient, and he give us an informed consent to publish this importante case.

**Figure 5:**
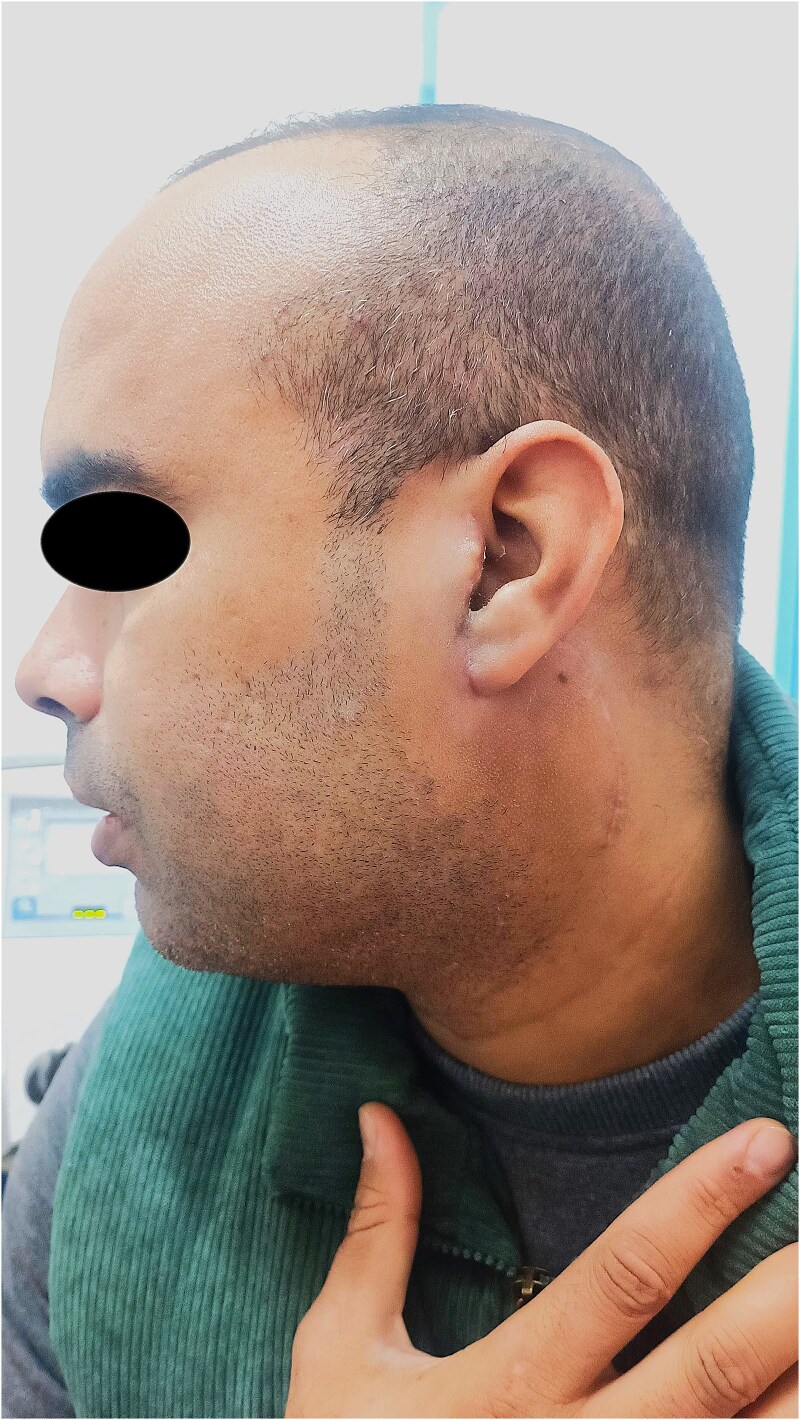
Postoperative photograph 6 months after surgery showing excellent healing and no signs of recurrence.

At 6-month follow-up, the patient remained asymptomatic, with no signs of recurrence or facial nerve dysfunction (Fig. 5).

## Discussion

Sebaceous lymphadenoma is a rare benign tumor of the salivary glands, accounting for ˂0.2% of all salivary gland neoplasms [1].

The vast majority of reported cases involve the parotid gland, predominantly in middle-aged adults, with no significant sex predilection [2].

Although the lesion is histologically distinctive, its clinical and radiological features often overlap with more common benign tumors, such as Warthin tumor and pleomorphic adenoma, making preoperative diagnosis challenging [3].

Clinically, sebaceous lymphadenomas typically present as slow-growing, painless masses. In our case, the patient presented with a 1-year history of a gradually enlarging, painless parotid swelling, without facial nerve involvement, a presentation consistent with most published reports [4, 5].

Imaging findings are generally nonspecific. In our case, the CT scan showed a well-defined, oval, enhancing lesion, which could easily be mistaken for a Warthin tumor or a low-grade mucoepidermoid carcinoma [6].

Histologically, sebaceous lymphadenoma is characterized by islands of benign epithelial cells with sebaceous differentiation embedded in a lymphoid-rich stroma, sometimes with germinal centers [7].

Immunohistochemistry plays an important role in confirming the sebaceous nature of the epithelial component, particularly in differentiating it from other tumors with a lymphoid background [8]. In our case, the diagnosis was definitively established by histopathology and supported by immunohistochemical analysis.

The treatment of choice is surgical excision, typically via superficial parotidectomy, with careful preservation of the facial nerve [9].

Recurrence is extremely rare, and malignant transformation has not been convincingly documented in the literature [10]. Our patient underwent an uneventful superficial parotidectomy and remained recurrence-free after 6 months, consistent with the benign and indolent nature of this tumor.

In conclusion, this case adds to the limited number of published reports and highlights the importance of considering sebaceous lymphadenoma in the differential diagnosis of parotid tumors, particularly when imaging findings are not pathognomonic. Awareness of this rare entity is essential to avoid over-treatment or misdiagnosis.

The main strength of this report lies in the complete diagnostic and therapeutic work-up, including radiological assessment, surgery with nerve preservation, and definitive histopathological diagnosis.
